# Population-Based Biobanking

**DOI:** 10.3390/genes15010066

**Published:** 2024-01-03

**Authors:** Wolfgang Lieb, Eike A. Strathmann, Christian Röder, Gunnar Jacobs, Karoline I. Gaede, Gesine Richter, Thomas Illig, Michael Krawczak

**Affiliations:** 1 Institute of Epidemiology and Biobank Popgen, Kiel University, University Hospital Schleswig-Holstein, Campus Kiel, 24105 Kiel, Germany; eike.strathmann@epi.uni-kiel.de (E.A.S.); c.roeder@email.uni-kiel.de (C.R.);; 2 German Centre for Lung Research (DZL), Airway Research Centre North (ARCN), 22927 Großhansdorf, Germany; kgaede@fz-borstel.de (K.I.G.); gesine.richter@iem.uni-kiel.de (G.R.); illig.thomas@mh-hannover.de (T.I.); 3 PopGen 2.0 Biobanking Network (P2N), Kiel University, University Hospital Schleswig-Holstein, Campus Kiel, 24105 Kiel, Germany; krawczak@medinfo.uni-kiel.de; 4 Institute for Experimental Cancer Research (IET), Kiel University, University Hospital Schleswig-Holstein, Campus Kiel, 24105 Kiel, Germany; 5 BioMaterialBank (BMB) North, Research Center Borstel, Leibniz Lung Center, 23845 Borstel, Germany; 6 Institute of Experimental Medicine (IEM), Division of Biomedical Ethics, Kiel University, University Hospital Schleswig-Holstein, Campus Kiel, 24105 Kiel, Germany; 7 Hannover Unified Biobank (HUB), Hannover Medical School, 30625 Hannover, Germany; 8 German Center for Lung Research (DZL), Biomedical Research in Endstage and Obstructive Lung Disease Hannover (BREATH), 30625 Hannover, Germany; 9 Institute of Medical Informatics and Statistics (IMIS), Kiel University, University Hospital Schleswig-Holstein, Campus Kiel, 24105 Kiel, Germany

**Keywords:** biobanking, biosample, biomarker, cohort study, clinical routine, phenotype, health data, sustainability, governance, public involvement

## Abstract

Population-based biobanking is an essential element of medical research that has grown substantially over the last two decades, and many countries are currently pursuing large national biobanking initiatives. The rise of individual biobanks is paralleled by various networking activities in the field at both the national and international level, such as BBMRI-ERIC in the EU. A significant contribution to population-based biobanking comes from large cohort studies and national repositories, including the United Kingdom Biobank (UKBB), the CONSTANCES project in France, the German National Cohort (NAKO), LifeLines in the Netherlands, FinnGen in Finland, and the All of Us project in the U.S. At the same time, hospital-based biobanking has also gained importance in medical research. We describe some of the scientific questions that can be addressed particularly well by the use of population-based biobanks, including the discovery and calibration of biomarkers and the identification of molecular correlates of health parameters and disease states. Despite the tremendous progress made so far, some major challenges to population-based biobanking still remain, including the need to develop strategies for the long-term sustainability of biobanks, the handling of incidental findings, and the linkage of sample-related and sample-derived data to other relevant resources.

## 1. Introduction

The standardized collection, storage, and retrieval of human biosamples for scientific use (jointly termed ’biobanking’) are essential components of modern biomedical research. Over the last two decades, there has been enormous progress and growth of these activities, not least because of the significant improvement of biosample storage and analysis technologies [1,2,3].

In this article, we present an overview of current activities in population-based biobanking, defined as the implementation and upkeep of large collections of human biosamples, either from patients with specific diseases, or from the general population. While prospective scientific cohort studies that collect biosamples together with clinical and physiological data have made a significant contribution in this regard, an increasing number of hospitals has also started to implement biobanking in clinical routine (‘hospital-based’ biobanking), and to link their collections to electronic health records and other sources of patient-related data [4,5].

We start this article with describing examples of large cohort studies and hospital-based biobanks, followed by an outline of research questions that can be addressed specifically well using population-based biobanks. Then, we describe national and international networking activities of the biobanking community, and close by describing some current and future challenges to population-based biobanking.

### 1.1. Population-Based Cohort Studies Collecting Biosamples

A significant contribution to population-based biobanking comes from large population-based cohort studies for which participants are drawn at random from the general population. These studies usually entail comprehensive medical assessments in dedicated study centers, have a longitudinal perspective, because they follow participants over years or even decades, and collect health-related information and biosamples repeatedly.

The enrichment of cohort studies by the systematic collection of biosamples started almost 50 years ago in the Framingham Heart Study (personal communication) and continued, in the mid-1980s, with, for example, the Augsburg MONICA surveys [6], amongst others. A more recent landmark project is the United Kingdom Biobank (UKBB) the concept of which was first laid out in 1999, and which was founded as a charitable company in 2003 [7]. The recruitment of participants into the UKBB started in 2006, and its original goal of 500,000 participants was reached in 2010 [8]. Data and biosamples (e.g., blood and urine) were collected in 22 assessment centers throughout the U.K. following a detailed protocol [8,9]. Biosamples were only minimally processed at the local examination sites, including centrifugation and intermittent storage until the end of the day, when samples were shipped to a central laboratory in Manchester for further processing and long-term storage at very low temperature [8,9]. A broad spectrum of genetic data has since become available in the UKBB as well, including whole-genome SNP genotypes as well as whole-exome and whole-genome DNA sequencing data from either the entire cohort or large parts of it [10]. Furthermore, several biomarkers of, for example, cardiovascular risk or kidney and liver function have been measured in UKBB participants and are available for research [11].

The German National Cohort (NAKO-Gesundheitsstudie, NAKO) is the largest population-based cohort study in Germany. Between 2014 and 2019, a total of 205,415 participants were recruited into the NAKO and comprehensively examined at one of 18 study centers [12]. Their clinical characterization included a variety of medical examinations and tests, standardized interviews and, in a subsample of 30,000 participants, a whole-body MRI [12]. A broad spectrum of biosamples has also been collected at the study centers, including serum, EDTA plasma, buffy coat, urine, stool samples, saliva, and nasal swabs [12]. Samples were taken from participants during their baseline visit, and were immediately centrifuged, aliquoted and stored intermittently. Roughly one third of the biosamples (32 aliquots per participant) have remained in the local storage facilities for back-up, whilst two thirds (72 aliquots) were transferred to a dedicated repository at the Helmholtz Center in Neuherberg, near Munich [13]. This central NAKO facility has a capacity of more than 20 million aliquots. Biosamples are usually shipped from the study centers to Neuherberg by a commercial transport service, employing a temperature data logger to document the sample temperature during the transport [13].

CONSTANCES is a nation-wide population-based cohort study with significant biobanking activities in France [14,15]. A total of 202,045 participants were randomly selected from the register of the National Pension Insurance Fund and underwent medical examination at one of 24 study centers in 21 metropolitan regions of France [14]. Systematic biobanking in CONSTANCES started in 2018 and includes urine, buffy coat, EDTA plasma, and lithium heparin plasma and serum samples. Plasma and serum are centrifuged locally and intermittently stored at +2/+8 °C [14]. Within 30 h after collection, samples are shipped to the Integrated Biobank of Luxembourg for automated, robot-assisted aliquoting and long-term storage in liquid nitrogen [14]. CONSTANCES currently aims at collecting samples from 83,000 participants.

The Finns have a rather distinct history and genetic make-up compared with other European populations. They were exposed to a strong population bottleneck about 120 generations ago, resulting in an enrichment of rare genetic variants and an increased prevalence of several inherited, mostly recessive diseases referred to as the Finnish Disease Heritage [16,17]. The FinnGen project combines a broad spectrum of biobanking activities from across the country, including hospital-based, private health care, and blood donor biobanks. The contributing centers process data and biosamples on different legal bases, from study-specific consent to broad consent [16]. One particular strength of FinnGen is that biosamples and biosample-derived data can be linked to longitudinal health registers that have been operational in Finland since 1969 [16]. Genome-wide SNP genotype data are available from more than 220,000 individuals, and a comprehensive genetic analysis of over 1,900 different diseases and clinical endpoints, drawing upon FinnGen resources, was published recently [16].

The Netherlands have a strong track record in establishing and maintaining large cohort studies, including the Rotterdam Study [18] and the Maastricht Study [19]. Between 2006 and 2013, a total of 167,729 individuals from the north of the country (approximately 10% of the regional population) were recruited into LifeLines, a longitudinal cohort study planned to run for at least 30 years and to cover three generations [20]. Its family-based design, in particular, offers unique research opportunities to LifeLines. The cohort has been comprehensively phenotyped, and several types of biosamples are stored for future analysis. The collected data come from different questionnaires and from a comprehensive assessment of the participants’ physical and mental health. One focus of LifeLines is on lifestyle factors and the exposome (including, for example, data on air quality and noise exposure), aimed at identifying modifiers of healthy aging [20,21]. All participants aged eight years or older are examined physically at one of 12 local centers, and biosamples (e.g., different fasting blood samples and 24-h urine) are taken locally and transported to the LifeLines laboratory in Groningen for central processing and long-term storage at −80 °C [20,21]. In specific sub-cohorts, additional biosamples (e.g., feces, hair, cord blood, and placental tissue) are also collected, and an MRI of the brain as well as a CT of the lungs are performed [21]. By April 2021, 165 participants had undergone whole-genome DNA sequencing [21], whilst SNP genotype data are available from about 50,000 participants [21].

The ‘All of Us’ project is a cohort study in the U.S. with a special focus on diversity and on the inclusion of minorities, who have hitherto been underrepresented in biomedical research [22,23]. The project collects biosamples and gathers a broad spectrum of health-related information, either directly from participants (via questionnaires and wearables), from electronic health records, or from additional sources such as drug prescriptions or health care claims. Notably, the project even collects geospatial environmental data [22]. ‘All of Us’ aims to ultimately include one million individuals, drawing upon 340 recruitment sites [22]. By November 2023, more than 739,000 participants had been recruited and more than 524,000 biosamples received [24].

The WHO IARC (International Agency for Research on Cancer) biobank maintains one of the largest biobanks for cancer research that comprises over 50 research projects, including 4 million biosamples from >370,000 members of the EPIC (European Prospective Investigation into Cancer and Nutrition) cohort [25]. EPIC is one of the largest population-based research projects focusing on the association between nutrition and incident cancer. Data and biosamples were collected in 23 study centers in 10 countries [26].

### 1.2. Hospital-Based Biobanking

In addition to large cohort studies, research biobanks have also been set-up by hospitals, for example, to collect left-over blood samples for DNA extraction and genetic analysis. Some countries have developed national strategies for such hospital-based biobanking. One of the first and hitherto largest initiatives has been the Electronic Medical Records and Genomics (eMERGE) network in the U.S. The participating centers extract and analyze DNA from patients and link the resulting genetic information to electronic health records. This allows researchers to address a variety of scientific questions about the impact of genetic variation on disease risk, and on clinical decision making [4,27]. Similar initiatives have been started in Europe as well, mainly in regions with well-established electronic health records, such as Scandinavia. The University Hospital of Copenhagen, for example, has stored left-over blood samples for research purposes since 2009 and, by 2020, biosamples from more than 420,000 patients were available in the associated Copenhagen Hospital Biobank (CHB) [5]. Analytes measured in these samples can be linked to a variety of national medical registries [5]. The CHB is also part of the Danish National Biobank, which stores and manages millions of samples from defined research projects, screening examinations, and from the medical treatment of Danish patients [28]. The FinnGen project mentioned above also comprises several hospital-based biobanks [16]. The University of Graz hosts one of the largest biosample repositories in Europe, holding around 20 million samples of many different tissue types [29]. Finally, various healthcare-integrated research biobanks have been set up by university hospitals in Germany, including Leipzig [30], Hannover [31], Lübeck [32], and Kiel [33], and by non-university research organizations, such the Helmholtz Society and the Leibnitz Society [34,35].

## 2. Scientific Value of Population-Based Biobanking

A fully comprehensive appraisal of the scientific value of population-based biobanking is beyond the scope of this article. However, there are certain research questions, highlighted below, that can be addressed specifically well by the use of biosamples collected on a population basis.

By design, population-based biobanks are ideally suited to define reference values for biomarkers. Determining its statistical distribution in the general population or in healthy individuals (as identifiable in well characterized cohorts) is an important step during the evaluation of a new biomarker and a prerequisite for judging its capacity to indicate prevalent or future disease [36]. If a sample collection is truly representative of the underlying population (which may not always be the case due to, for example, low participation rates [12,14,37]), it also allows estimation of the prevalence of the disease that is diagnosed by the biomarker. Anyhow, even if a sample collection is biased and therefore not perfectly suitable for parameter estimation, it can still highlight differences in the statistical distribution of a biomarker between, say, patients and the general population.

In combination with sufficiently rich clinical and physiological data, population-based and hospital-based biobanks can help to identify the biochemical and molecular correlates of human phenotypes, particularly of binary disease states and continuous health parameters, such as blood pressure [38], kidney function [39], and BMI [40]. Thus, population- and hospital-based biobanks have been particularly useful to genome-wide associations studies (GWAS) of the genetic architecture of common complex diseases [41]. The powerful combination of biosamples with associated data has also allowed researchers to investigate the extent and nature of gene-environment (lifestyle) interactions in relation to various diseases [42].

One particular strength of biosample collections embedded in large cohort studies is the prospective design of the latter and the repeated long-term assessment of participants. Surveying initially healthy individuals for many years or even decades provides an unparalleled opportunity for researchers to relate biomarkers to new-onset (incident) diseases [43] and to organ function decline [44]. Biosamples obtained in a clinical context, on the other hand, usually come from patients and are therefore an invaluable resource for case-control studies, including GWAS. Moreover, biomarkers measured during clinical care are likely to be informative about diagnostic performance (including disease staging), disease progression, and treatment response, and about clinical complications and mortality [36,45].

## 3. Biobanking Networks

Several national and international networks have been initiated over the last 15 years to connect and professionalize existing biobanks. Most importantly, a European Biobanking and BioMolecular Resources Research Infrastructure (BBMRI) was devised in 2007 [46] and granted legal status as a European Research Infrastructure Consortium (ERIC) in 2013 [47]. ERIC is a generic organizational form that allows EU member states to jointly establish and operate research infrastructures of overarching interest. ERIC status has facilitated the establishment of so-called BBMRI-ERIC ‘nodes’ that bring together research biobanks at a national level, thereby promoting the development of a coherent European biobank network [48]. BBMRI-ERIC lends extra prominence to its partners, their services and their expertise on a non-commercial basis. Key areas supported by BBMRI-ERIC include (1) sample processing and storage quality management [49], (2) ethical, legal and social issues of biobanking (Common Service ELSI) [50,51], (3) data safety and data protection, aiming at a code of conduct for health research [52], and (4) the Common Service IT to improve the online visibility and accessibility of biobanks [53]. To all of these ends, BBMRI-ERIC offers various tools and services for the communication with and among biobanks, including the BBMRI-ERIC Biobank Directory, Sample Locator and Sample Negotiator [54,55].

### Activities at the National Level: Germany as an Example

In Germany, biobanking initiatives in the academic sector are being coordinated by the German Biobank Node (GBN), the German Biobank Alliance (GBA), and the Technology and Methods Platform for Networked Medical Research (TMF) [56,57]. The TMF is a semi-state umbrella organization that has played an important role, for almost 25 years, in networking and supporting diverse health research-related activities in Germany, focusing upon IT, data protection, ELSI, and biobanking. From 2011 to 2016, the German Federal Ministry of Education and Research (BMBF) funded five university hospitals to establish ‘centralized Bio-Material Banks (cBMB)’. The aim of the program was to establish a cooperative platform for individual biobanks located at the chosen sites and, thereby, to lay the foundation for a nation-wide network of research biobanks. In conjunction with the cBMB program, the German National Biobank Symposium was established as an annual meeting of the national biobank community, facilitating the exchange of experiences and perspectives on the organizational, technical and legal-ethical aspects of biobanking.

The above-mentioned GBN has become the key link between the German and European biobanking community. It was originally established and funded within the cBMB program as its national focal point. Today, GBN acts as the German node of BBMRI-ERIC and has been responsible for the foundation of the GBA in 2017 in continuation of the cBMB program [57]. Since then, GBA has turned into a national network comprising nearly all research biobanks maintained at German university hospitals. The GBA develops standards, infrastructure and tools for IT, quality management, stakeholder engagement, public outreach and training, and thereby contributes greatly to the professionalization and harmonization of biobanking in Germany [57].

## 4. Challenges to Population-Based Biobanking

Population-based biobanking is also facing a number of specific challenges. First and foremost, samples should undoubtedly be used most effectively, and a maximum of information extracted from them. Because of the tremendous technological progress in the field of molecular biology, whole-genome DNA sequencing and other OMICs profiling can be performed today, even for large numbers of individuals [1,3]. However, the costs of producing such data for an entire cohort or relevant sub-cohort can still add up to several million Euros, particularly for large studies comprising tens or sometimes hundreds of thousands of participants. These financial demands represent a great challenge to host institutions and funding agencies alike. One possible solution to the problem would be a more intense collaboration with industry as pursued, for example, by the UKBB [58] and FinnGen [59]. Its attractiveness notwithstanding, however, the large-scale involvement of industry in publicly funded cohort studies and biorepositories is likely to create new challenges. For example, whereas such collaboration, of course, would have to be covered by the consent given by participants, it may still be viewed critically. We previously observed that while the overall willingness to donate data for academic research is high among German outpatients, only a minority is willing to support collaborations with industry [60,61]. Concerns include fears that such research may not serve the common good, that data protection by commercial companies may not be sufficient, and that companies benefit economically from using the samples [60].

Second, the scientific value of biosamples depends critically upon the scope and nature of the health-related information that can be linked to them. Such information may be obtained directly from the sample donor (e.g., through questionnaires, on-site examinations, or wearables), from hospital information systems, from health insurers and pension funds, or from disease or mortality registries. The ease of access to such secondary data varies widely across Europe. While medical data linkage is well-established in Scandinavia [5,16], it is cumbersome in other European countries, including Germany. Moreover, the IT and data management infrastructure required for a biobank to be able to participate in data linkage is demanding and often simply not available.

Another key issue is the sustainability of biobanks. Storing millions of samples over years or even decades at low temperature incurs both investment and maintenance costs. Long-term storage, in particular, is likely to render biosamples scientifically highly valuable because decades of follow-up data can be combined with the results of modern (molecular) analysis techniques, but is also very cost-intensive. On the other hand, it is very difficult to precisely quantify the costs of storing, say, one sample or aliquot of a particular type of tissue for one year. In the same vein, the costs of the acquisition of a sample may vary substantially, depending upon whether it is obtained from a cohort study, hospital-based study, or a basic science project. Therefore, viable sustainability concepts are critical for the long-term survival of biobanks, and will become even more so in view of continuously increasing energy costs. Although sustainability has been a subject of great interest in the past, and led some biobanks to introduce storage fees, to the best of our knowledge, there is no generally accepted cost model for biobanking yet.

Ethically and legally acceptable biobanking requires efficient governance structures to regulate the use and access of biosamples. There has been considerable progress in this regard, and most large cohort studies and academic biobanks have implemented easily accessible and transparent means to facilitate (and actively encourage) the research use of their samples and data. This usually involves websites with detailed information about the available resources and application procedures. A prime example in this regard is the UKBB, which has received and processed an average of 561 research proposals per year between 2020 and 2022 [62]. In contrast to many other cohort studies that mainly benefit a small core of key investigators, the UKBB is extensively used by the global scientific community and has generated an unparalleled number of scientific publications during its existence.

An ever more pressing challenge to research biobanks is the handling of incidental findings. With the advent of whole-exome and whole-genome DNA sequencing, the chance of making incidental findings in biobank-based research has increased substantially. We cannot discuss in much detail how such findings are best dealt with, not least because detailed recommendations to this effect have been published before in the clinical context [63], for hospital-based biobanks [64] and for population-based studies [65]. Nevertheless, named recommendations are often difficult to follow for a number of reasons: Commonly, incidental findings are defined as being (i) unrelated to the primary analysis purpose, (ii) clinically relevant, and (iii) actionable [65]. For many biomarkers, and for genetic variants in particular, the fulfillment of (ii) and (iiii) is, however, not always clear and may even change over time, depending upon scientific progress. This poses a great challenge to biobanks because their long-term design implies that several years may pass until an incidental finding is made. What is more, by the time a biobank sets out to report a finding to a participant, the latter may have forgotten about their involvement and are suddenly confronted with a serious health issue. Moreover, if the possible consequences of a clinically relevant and actionable mutation are already difficult to communicate in the health care context, this is even more true for cohort studies and biobanks.

Since human data and biosamples should always be used for medical research in the most comprehensive and efficient way, the legal possibility of sharing them at both the national and international level is another relevant issue for biobanks. Within the EU, the GDPR (General Data Protection Regulation) defines the legal framework for the acquisition, handling, and storage of personal data and, thus, of research data obtained from, or together with, biosamples. Sharing of such data is allowed under the condition that there is a legal basis for this, which, in most instances, will be the informed consent of the data subjects. As regards the transfer of data to countries outside the EU, the GDPR foresees two main mechanisms of legal permission, namely an adequacy decision by the EU (Art 45, GDPR) or a guarantee for the provision of “appropriate safeguards” (Art. 46).

In the recent past, broad consent has taken on great importance as a legal framework in medical research. As the name suggests, with broad consent, patients are informed about general research goals rather than the specific research agendas pursued with their data and biosamples in terms of, for example, the diseases covered. Broad consent also allows data and biosamples to be stored for long periods of time because many research questions for which these resources may turn out useful will likely only arise in the future. In return, broad consent is usually linked to the mandatory oversight of research projects by ethics boards and use and access committees, as well as information about ongoing research being continuously provided to data subjects and biosample donors [66].

Finally, patient and public involvement plays an increasingly important role in science in general, and in biobank-based research in particular [67]. Informing donors and the general public about research with human biosamples should be imperative, given that samples and data are voluntarily donated and that most biobanking activities are publicly funded. Many large biorepositories and cohort studies already maintain websites that provide comprehensive information about research projects and results, or they use newsletters to inform participants. In addition, new formats of information provision have been implemented, including movies, podcasts [68,69], and comics [70,71]. Not least, these innovations were motivated by the fact that classical ways of informing patients and participants, particularly in a clinical context, may not lead to a sufficient understanding of the topic at hand by the addressees [72,73]. Moreover, a greater variety of information media allows researcher to account more for people’s rather differentiated view of biobanking, as revealed by recent research [60,74,75].

Other important facets of patient and participant involvement include the external evaluation of the information material and consent forms used, and a better consideration of the donors’ views and motivations in the planning and governance of biobank-based research [74].

## 5. Concluding Remarks

Population- and hospital-based biobanking have grown enormously over the last two decades. In fact, biobanking has become an integral part of many large cohort studies, and dedicated research biobanking is also taking place in many hospitals. Some countries have even developed and implemented nationwide biobanking initiatives. All these activities have greatly contributed to the scientific progress in biomedicine. For example, our understanding of the genetic basis of common diseases has improved much through the analysis of biosamples collected in either clinical settings or large cohort studies. On the other hand, some important challenges to biobanking remain, including a lack of financial and organizational sustainability, an obligation to make the most effective use of the samples, the need to link samples to relevant secondary data sources, and the possibility of incidental findings. These challenges notwithstanding, population-based and hospital-based biobanking will likely continue to represent an essential foundation of biomedical research.

## Data Availability

No new data were created or analyzed in this study. Data sharing is not applicable to this article.
